# Angiogenin Reduces Immune Inflammation via Inhibition of TANK-Binding Kinase 1 Expression in Human Corneal Fibroblast Cells

**DOI:** 10.1155/2014/861435

**Published:** 2014-04-17

**Authors:** Seung Hoon Lee, Kyoung Woo Kim, Kyong-Mi Min, Kyu-Wan Kim, Soo-Ik Chang, Jae Chan Kim

**Affiliations:** ^1^Department of Ophthalmology, College of Medicine, Chung-Ang University Hospital, 224-1 Heukseok-dong, Dongjak-Gu, Seoul 156-755, Republic of Korea; ^2^Department of Medicine, Graduate School, Chung-Ang University, Seoul, Republic of Korea; ^3^Department of Biochemistry, Chungbuk National University, 52 Naesudong-ro, Heungdeok-gu, Cheongju, Chungbuk 361-763, Republic of Korea

## Abstract

Angiogenin (ANG) is reportedly multifunctional, with roles in angiogenesis and autoimmune diseases. This protein is involved in the innate immune system and has been implicated in several inflammatory diseases. Although ANG may be involved in the anti-inflammatory response, there is no evidence that it has direct anti-inflammatory effects. In this study we sought to determine whether ANG has an anti-inflammatory effect in human corneal fibroblasts (HCFs) exposed to media containing tumor necrosis factor-alpha (TNF-**α**). We found that ANG reduced the mRNA expression of interleukin-1 beta (IL-1**β**), -6, -8 and TNF-**α** receptors (TNFR) 1 and 2. In contrast, ANG increased the mRNA expression of IL-4 and -10. Protein levels of TANK-binding kinase 1 (TBK1) were reduced by ANG in HCFs treated with TNF-**α**. Moreover, ANG diminished the expression of IL-6 and -8 and monocyte chemotactic protein- (MCP-) 1. The protein expression of nuclear factor-**κ**B (NF-**κ**B) was downregulated by ANG treatment. These findings suggest that ANG suppressed the TNF-**α**-induced inflammatory response in HCFs through inhibition of TBK1-mediated NF-**κ**B nuclear translocation. These novel results are likely to play a significant role in the selection of immune-mediated inflammatory therapeutic targets and may shed light on the pathogenesis of immune-mediated inflammatory diseases.

## 1. Introduction

Ocular inflammation is one of the main causes of blindness and visual disturbance. A number of ocular inflammatory diseases cause visual impairment and chronic immune-mediated inflammation in the eye can lead to blindness [1–4]. Inflammation is a main component in the pathophysiology of several ocular diseases including corneal and autoimmune diseases. It has generally been accepted that inflammatory cytokines and chemokines are significantly increased in many ocular diseases and immune rejection of corneal transplantation [5–8].

The corneal stroma is a thick, transparent layer that is frequently subjected to the inflammatory response [9]. Chronic inflammation of corneal fibroblasts causes corneal scarring, neovascularization, edema, opacity, injury, ulceration, and ultimately impaired vision and blindness [4, 9–11]. Human corneal fibroblasts (HCFs) in the corneal stroma perform a significant function in the control of local immune and inflammation [12]. It has been widely reported that corneal fibroblast cells act as sentinel cells of the immune system and participate in the regulation of stromal inflammation through the production of cytokines and chemokines [13–15]. Corneal fibroblast cells secrete proinflammatory cytokines and chemokines including interleukin (IL)-6 and IL-8 in response to external inflammatory stimuli [12, 15, 16].

Tumor necrosis factor-*α* (TNF-*α*) is a cytokine involved in the inflammatory response that binds two receptors and tumor necrosis factor receptors (TNFR) 1 and 2. It has been demonstrated that TNF-*α* plays a critical role in corneal inflammation [17]. TNF-*α* promotes nuclear factor-*κ*B (NF-*κ*B) translocation, and the NF-*κ*B signaling pathway, which is found in all mammalian cell types, comprises several genes that influence immune and inflammatory responses [18, 19]. NF-*κ*B is activated by phosphorylation of the regulatory protein I-kappa-B (I*κ*B) and TANK-binding kinase 1 (TBK1). TBK1 promotes NF-*κ*B activation and may act downstream of the NF-*κ*B transcriptional pathway [20, 21]. It is well documented that TBK1 inhibitors decrease inflammation [22].

Angiogenin (ANG) is a 14.4 kDa single chain protein containing 123 amino acids. Several studies have demonstrated its function with respect to immunity. This protein is a component of tears and protects the ocular surface as it is an antimicrobial peptide [23, 24]. ANG mRNA expression and ANG protein concentrations in serum are increased during the inflammatory response [25, 26]. Serum ANG was found to be increased in patients with inflammatory bowel disease, and ANG mRNA expression was shown to be elevated by TNF-*α* and IL-1*β* [27, 28]. ANG was also shown to have bactericidal activity [29] and has been reported to be a microbial recognition protein related to innate immunity [30]. This evidence suggests that ANG may play a role in modulating the inflammatory response.

We hypothesized that ANG has an inhibitory effect on the inflammatory response in the ocular surface. The aim of this study was to determine whether ANG has an anti-inflammatory effect in HCFs treated with TNF-*α*. Thus, we attempted to clarify the molecular activity of ANG underlying its inhibition TNF-*α* mediated transduction, which involves the production of TBK1 and nuclear translocation of NF-*κ*B.

## 2. Methods

### 2.1. Isolation and Primary Culture of Human Corneal Fibroblast Cells

Human corneal donor tissues were obtained during penetrating keratoplasty. The corneal epithelium was eliminated and then the corneal fibroblast cells were detached from explant tissue. The corneal tissues were rinsed with phosphate-buffered saline (PBS) mixed with 5% penicillin-streptomycin. After the corneal epithelium was eliminated, the corneal stroma was cut into explants of approximately 1 mm^3^. Each piece of explant was placed on a culture dish and immersed in culture medium for one week. The HCFs were then subcultured by trypsin digestion. HCFs were cultured in alpha-minimum essential medium (*α*-MEM) (Invitrogen-Gibco, USA) containing 10% FBS and 1% penicillin-streptomycin. The cells were maintained at 37°C under 5% CO_2_ and used for experiments after three to five passages. The study protocol and informed consent were approved by the institutional review board of the Chung-Ang University Hospital. This study conformed to the tenets of the Declaration of Helsinki.

### 2.2. Cell Treatment

HCFs were cultured in six-well plates for three days. They were washed twice with PBS. The medium of confluent corneal fibroblast cells was changed to serum-free MEM for one day before treatment. The cells were treated with TNF-*α* purchased from PROSPEC (20 ng/mL) for eight hours, and with or without ANG (2 *μ*g/mL) at the last 30 minutes of incubation with TNF-*α*. ANG was obtained from the Department of Biochemistry at Chungbuk National University and the identity of the purified ANG has been confirmed by western blotting with ANG specific antibodies by methods described in a previous report [31]. The biological activity of the purified ANG has also been confirmed by its nuclear translocation in HUVE cells by procedure described in detail [31]. The purification and endotoxin levels of recombinant ANG expressed in* E. coli* are shown in Supplementary Figures  1 and 2 (see the Supplememtery Material available online at http://dx.doi.org/10.1155/2014/861435). The cells were then collected for total RNA isolation and protein extraction.

### 2.3. RNA Isolation and Real-Time RT-PCR

Total RNA was isolated from cultured HCFs using FavorPrep Tri-RNA reagent, according to the manufacturer's protocols. The quantity and quality of the RNA were determined using a NanoDrop ND-1000 spectrophotometer (ND-1000, Nano-Drop Technologies, Inc. Wilmington, DE, USA). Single-stranded complementary DNA (cDNA) was synthesized from 500 ng of total RNA using a cDNA synthesis kit (Takara Bio Inc., Otsu, Japan). Real-time RT-PCR was conducted using the CFX96 Real-Time PCR Detection System (Bio-Rad, Hercules, CA, USA) in a total volume of 20 *μ*L containing 10 *μ*L of SYBR Premix Ex Taq (Takara Bio Inc., Otsu, Japan), diluted cDNA template, and forward and reverse primers. The primer sequences and product size are listed in Table 1. The PCR amplification for selected genes was run for 40 cycles. Gene expression was analyzed by real-time reverse transcriptase polymerase chain reaction (RT-PCR). Real-time PCR quantification was done in triplicate for each sample and the mean was calculated. Expression levels were analyzed by RT-PCR using values of glyceraldehyde-3-phosphate dehydrogenase (GAPDH) as a reference.

### 2.4. Immunodot Blot Assay

The expression of 42 human cytokines and chemokines was assessed using a commercially available cytokine assay (RayBio Human Cytokine Antibody Array 3, RayBiotech, Norcross, GA, USA) that utilizes membrane-bound cytokine-specific antibodies to assess the presence of several cytokines in biological fluids. The analysis was conducted according to the manufacturer's instructions. Briefly, membranes were blocked for 30 minutes and then incubated with HCFs culture supernatant for two hours at room temperature. The membranes were washed with Wash Buffer I three times for five minutes each and then with Wash Buffer II twice for five minutes each. After washing, the membranes were incubated with a biotin-conjugated antibody mix for two hours, and then streptavidin-conjugated peroxidase was added for two hours at room temperature. The membranes were subsequently washed thoroughly and exposed to chemiluminescence. The membranes were visualized using the ECL Plus detection system and ChemiDoc XRS (Bio-Rad Laboratories, Inc., Berkeley, CA, USA). The densities for individual spots were calculated using ImageJ software (Wayne Rasband, National Institutes of Health, USA). The relative expression ratio was determined by subtraction of the background signal and comparison with positive controls on the membrane. Positive controls visible within each array were used for comparison.

### 2.5. Nuclear and Cytosolic Protein Extractions

HCFs were washed and scraped with cold PBS. The cells were lysed in buffer A (10 mM HEPES (pH 7.9), 10 mM KCl, 0.1 mM EDTA, 0.1 mM EGTA, 1 mM DTT, 0.5 mM PMSF, and 5 *μ*g/mL Leupeptin) and left on ice for 15 min. After 10% NP-40 was added to the sample, the cytosolic fraction was collected by centrifugation at 14,000 rpm for 5 min at 4°C. The nuclear fraction was resuspended in buffer C (20 mM HEPES (pH7.9), 0.4 n NaCl, 1 mM EDTA, 1 mM EGTA, 1 mM DTT, 1 mM PMSF, and 10 *μ*g/mL Leupeptin) and left on ice for 30 min, then the nuclear fraction was collected by centrifugation at 14,000 rpm for 5 min at 4°C.

### 2.6. Western Blot Analysis

Nuclear proteins and total cell lysates were separated by 10% sodium dodecyl sulfate polyacrylamide gel electrophoresis (SDS-PAGE) and electrophoretically transferred to a polyvinylidene fluoride membrane (PVDF; Merck Millipore, Billerica, MA, USA) at 100 V (1 h) in buffer containing 0.3% Tris, 1.4% glycine, and 20% methanol using a wet-blotting apparatus (Mini-PROTEAN Tetra cell; Bio-Rad, Hercules, CA, USA). The PVDF membrane containing the transferred proteins was blocked with 5% BSA in PBS for one hour at room temperature. Primary monoclonal antibodies against human TBK1 (Abcam, Inc.) and NF-*κ*B (Bioworld Technology, Inc.) diluted in PBS (1 : 1000) were applied to the PVDF membrane and incubated overnight at 4°C. Secondary antibodies diluted in PBS (1 : 2000) were subsequently applied to the PVDF membrane and incubated for 1 h at room temperature. The PVDF membrane was washed four times (10 min each) with Tris-buffered saline (TBS; 50 mM Tris HCl pH 7.5, 150 mM NaCl) containing 0.1% Tween 20. The binding of specific antibodies was visualized using an enhanced chemiluminescence western blotting detection kit (Pierce Biotechnology, Inc., Rockford, IL, USA). Densitometric quantification of the immunoblot was carried out using ImageJ software. The value of each band was normalized to *β*-actin or lamin.

### 2.7. Immunocytochemistry

The HCFs cultured on glass slides were treated with TNF-*α* (20 ng/mL) for eight hours. Cells were also treated with or without ANG (2 *μ*g/mL) for 0.5 hours. Cells were then fixed in 4% paraformaldehyde for 15 min at room temperature. After being permeabilized by incubation with 0.5% Triton X-100 for 15 min at room temperature, the slides were incubated with anti-NF-*κ*B (diluted to 1 : 50 in PBS, Bioworld Technology, Inc.) for 1 h at room temperature. Glass slides were incubated with secondary antibody for 1 h at room temperature. At each step slides were washed three times (5 min each) with PBS. Cover slips were mounted on the slides using Vectashield (Vector Laboratories, Burlingame, CA, USA) containing 40,6-diamidino-2-phenylindole (DAPI).

### 2.8. Statistical Analysis

Data are expressed as the mean ± standard error (SE). Statistical analysis of three separate experiments was conducted using one-way ANOVA followed by a post hoc pairwise comparison adjusted with a Bonferroni correction. Statistical analyses were performed using SPSS software version 19.0 (SPSS Inc., Chicago, IL, USA). Differences were considered statistically significant at *P* < 0.05.

## 3. Results

### 3.1. ANG Inhibits mRNA Expression of Proinflammatory Cytokines and Promotes mRNA Expression of Anti-Inflammatory Cytokines in HCFs

**Figure 1:**
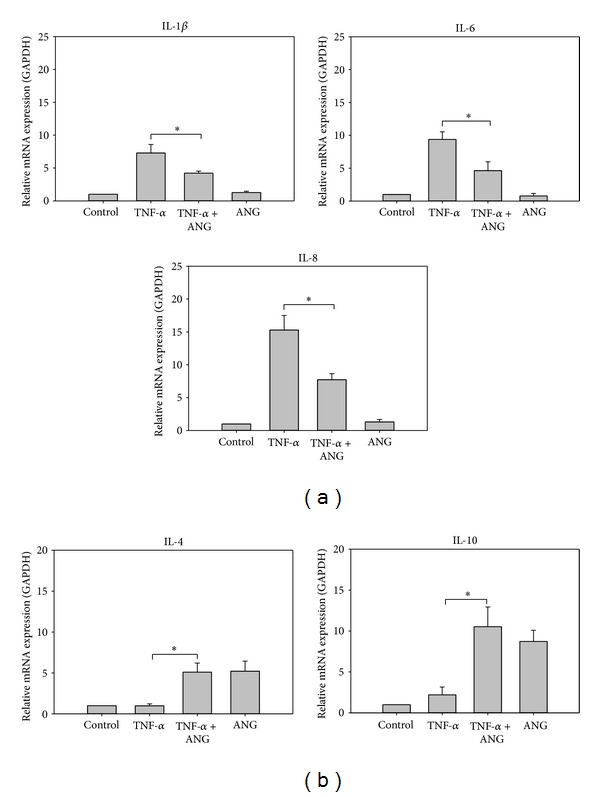
Real-time PCR analyses of proinflammatory cytokines and anti-inflammatory cytokines in HCFs. (a) The relative level of IL-1*β*, -6, and -8 mRNA was diminished by ANG treatment. ANG treatment alone did not affect the mRNA expression of proinflammatory cytokines. (b) The relative expression of IL-4 and -10 mRNA was increased by ANG treatment. ANG treatment alone increased the mRNA expression of IL-4 and -10. The experiments were performed in triplicate (*P < 0.05).

In order to determine whether ANG can reduce the inflammatory response in HCFs, TNF-*α* (20 ng/mL, 8 h) was added to the culture media and then cells were cultured in the presence or absence of ANG (2 *μ*g/mL, 30 min). Real-time PCR was conducted to investigate the effects of ANG treatment on the mRNA expression of proinflammatory (IL-1*β*, -6, and -8) and anti-inflammatory cytokines (IL-4 and -10). The expression of proinflammatory cytokines (IL-1*β*, -6, and -8) induced by TNF-*α* treatment was reduced significantly in cells treated with ANG (Figure 1(a)). ANG treatment alone did not exert influence on the mRNA expression of proinflammatory cytokines. Moreover, the mRNA expression of anti-inflammatory cytokines (IL-4 and -10) was increased significantly after ANG treatment (Figure 1(b)). ANG treatment alone increased the mRNA expression of IL-4 and -10.

### 3.2. ANG Suppresses the Expression of Inflammatory Cytokines and Chemokines in HCFs

**Figure 2:**
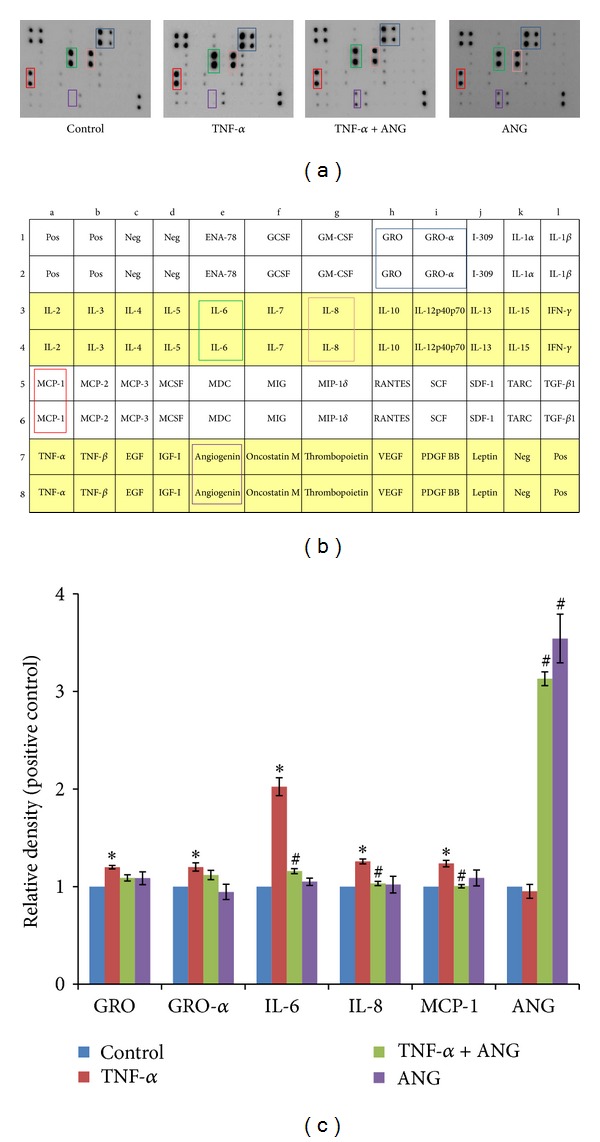
Inflammatory cytokine profiles in HCF culture medium. (a) Treatment with TNF-*α* (20 ng/mL) resulted in amplification of five inflammatory cytokines and chemokines (GRO and -*α*, IL-6 and -8, and MCP-1). Treatment with ANG (2 *μ*g/mL) resulted in reduction of these inflammatory cytokines and chemokines. Treatment with ANG alone only induced upregulation of ANF area. (b) Custom human growth factor antibody array map. Pos: positive control; Neg: negative control. (c) Relative density of inflammatory cytokines and chemokines. The signal intensities were observed and quantified using a chemiluminescence imaging device. The values presented in the bar graph are the mean ± SE from triplicate experiments (*P < 0.05 versus control cells; ^#^ *P* < 0.05 versus TNF-*α* treated cells). GRO: growth regulated oncogene; MCP: monocyte chemotactic protein.

Immunodot blot assays were conducted to determine whether ANG decreases inflammatory cytokines and chemokines in media. Treatment with TNF-*α* promoted the expression of inflammatory cytokines and chemokines such as IL-6 and -8, growth-related proteins (GRO), growth-related proteins-alpha (GRO-*α*), and monocyte chemotactic protein- (MCP-) 1. Production of these cytokines and chemokines was downregulated in the presence of ANG, but expression of ANG was upregulated by ANG treatment (Figures 2(a) and 2(b)). We detected significant differences when comparing media before and after ANG treatment with respect to the presence of IL-6 and -8, MCP-1, and ANG (Figure 2(c)). ANG treatment alone increased only ANG expression. The expression of another cytokines was not affected by ANG treatment alone.

### 3.3. ANG Suppresses TNFR mRNA Expression and TBK1 Production

**Figure 3:**
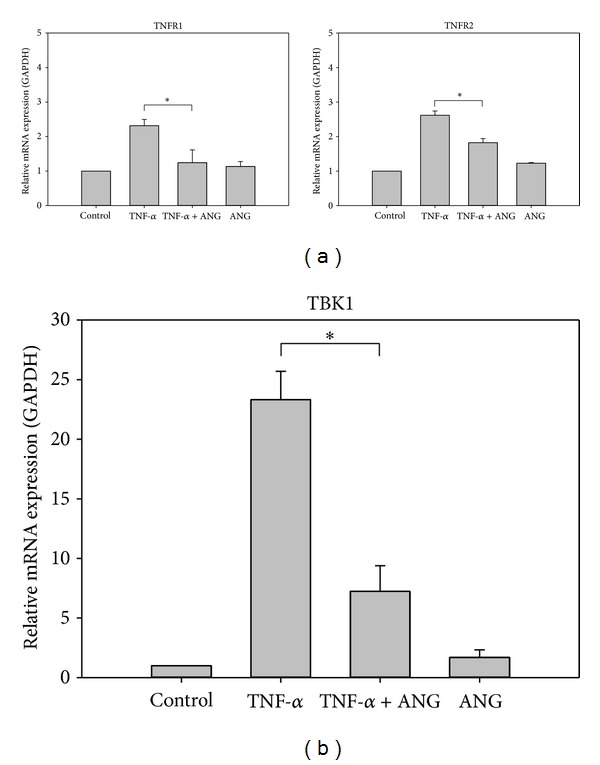
Real-time PCR analysis of TNFR1 and 2 and TBK1 in HCFs. (a) The relative level of TNFR1 and 2 mRNA was diminished by ANG treatment. (b) The relative level of TBK1 mRNA was diminished by ANG treatment. The experiments were performed in triplicate (*P < 0.05).

**Figure 4:**
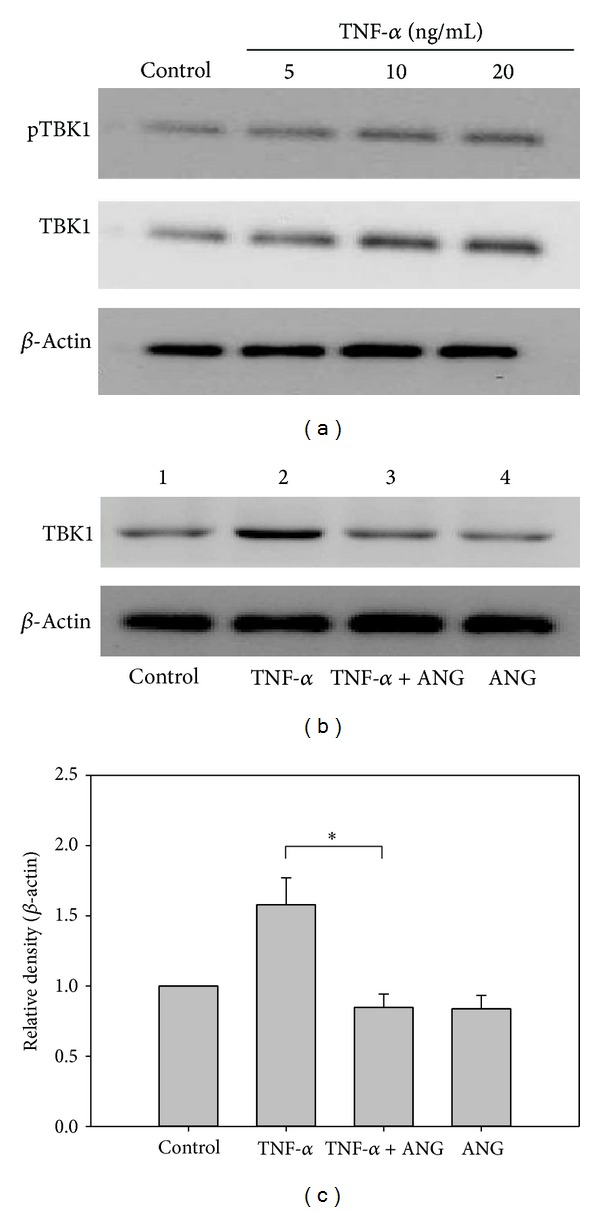
Western blot analyses of TBK1 protein expression and TBK1 phosphorylation (pTBK1). (a) TNF-*α* treatment increased the expression of TBK1 and TBK1 phosphorylation in HCFs. (b) After ANG treatment, TBK1 protein expression was decreased in HCFs. ANG treatment did not affect TBK1 protein expression. (c) Densitometric analysis of the relative ratio of TBK1 in cells treated with TNF-*α* only to those treated with TNF-*α*+ANG. The experiments were performed in triplicate (*P < 0.05).

Real-time PCR was performed to determine whether ANG decreases TBK1 and TNFR1 and 2. TNF-*α* treatment increased the mRNA expression of TNFR1 and 2, but a significant downregulation was noted after ANG treatment (Figure 3). ANG treatment alone did not affect the mRNA expression of TBK1 and TNFR1 and 2. Western blot analysis showed that ANG reduces the expression of TBK1. TNF-*α* treatment produced a dose-dependent increase in TBK1 phosphorylation and increased TBK1 expression. After ANG treatment, TBK1 expression was decreased (Figure 4). ANG treatment alone has little effect on the expression of TBK1.

### 3.4. ANG Inhibits Nuclear Translocation of NF-*κ*B

**Figure 5:**
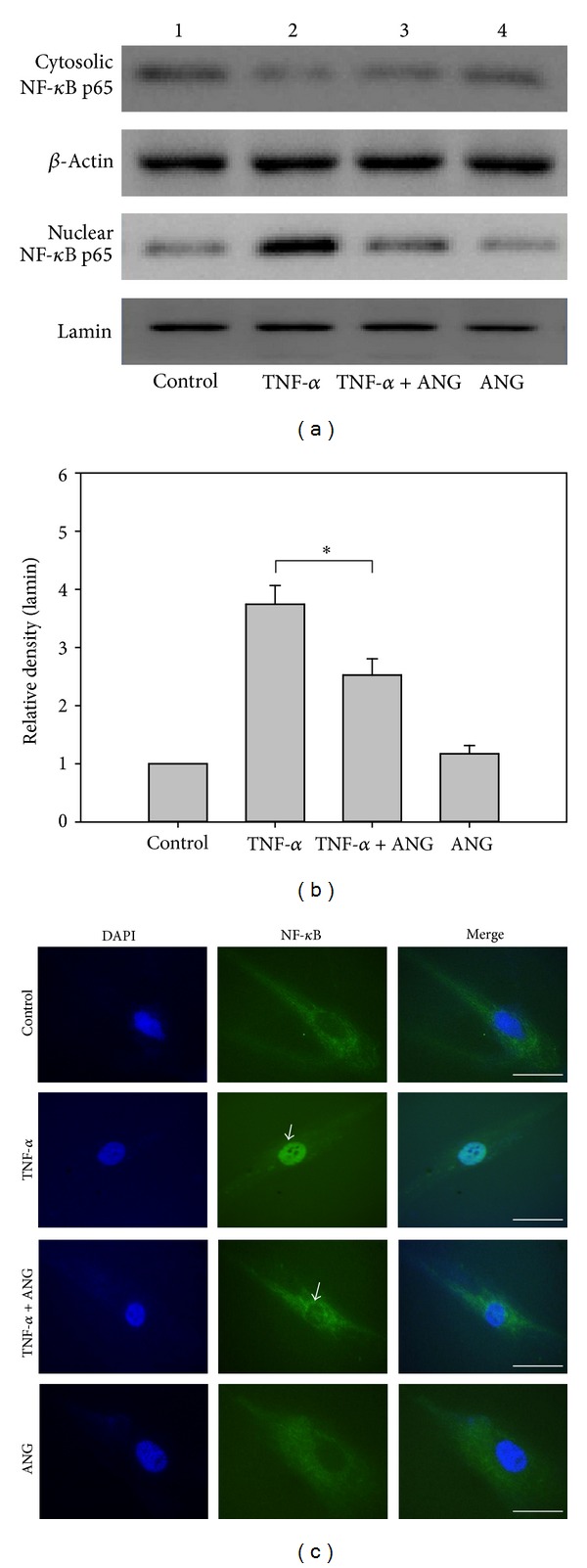
Western blot analyses of NF-*κ*B in the nucleus and cytoplasm. (a) NF-*κ*B nuclear translocation induced by TNF-*α* was diminished after ANG treatment. ANG treatment alone did not affect NF-*κ*B nuclear translocation. (b) Densitometric analysis of the relative ratio of NF-*κ*B in cells treated with TNF-*α* alone to those treated with TNF-*α*+ANG. The experiments were performed in triplicate (*P < 0.05). (c) Immunocytochemistry of NF-*κ*B in the nuclei and cytoplasm of HCFs. After treatment with ANG, the cells were fixed and then labeled with an anti-NF-*κ*B antibody. Immunofluorescent images at higher magnification demonstrate attenuation of the expression of NF-*κ*B in nucleus after treatment with ANG ((c) arrow) in cells treated with TNF-*α* (scale bar, 100 *μ*m).

HCFs were either cultured with TNF-*α* (20 ng/mL, 8 h) or treated with ANG (2 *μ*g/mL, 0.5 h) to examine whether ANG inhibits nuclear translocation of NF-*κ*B. The cells were subjected to immunofluorescent localization of NF-*κ*B. Treatment with TNF-*α* induced the translocation of NF-*κ*B from the cytosol to nucleus. However, the presence of ANG inhibited the translocation of NF-*κ*B (Figure 5). ANG treatment alone did not influence the expression of NF-*κ*B in nucleus.

## 4. Discussion

It is generally believed that ANG is an angiogenic molecule, inducing angiogenesis, cell migration, proliferation, and tumor growth [32–36]. Thus, previous studies have focused on its role in a possible antiangiogenin-based cure for cancer [37–39]. However, ANG is known to function as an antimicrobial peptide and is related to diverse inflammatory diseases and innate immunity [23, 28, 40–42]. Although ANG may have anti-inflammatory activity, there is currently no evidence in the literature to support this notion. In this study, we investigated the effect of ANG on inflamed HCFs and discovered a previously unidentified function of ANG in the anti-inflammatory mechanism.

The most substantial finding in our study is that ANG reduced the TBK1-mediated inflammatory response induced by TNF-*α* treatment. TBK1 is a member of the I*κ*B kinase family and is involved in the inflammatory response as it is related to NF-*κ*B activation [21]. TBK1 also has the ability to modulate the expression of IL-6 and TNF-*α* [43, 44]. It has been widely demonstrated that TBK1 plays a critical role in inflammatory diseases [45–47]. We have shown that ANG treatment decreased the expression of TBK1 in inflamed HCFs, suggesting that ANG may have possibility of anti-inflammatory activity.

It has been suggested that TNF-*α* induces the production of proinflammatory cytokines and chemokines including IL-6 and -8 and MCP-1 [48, 49]. These cytokines and chemokines are known to cause corneal inflammation [12, 50–54]. IL-6 produced by T cells and macrophages act as a proinflammatory cytokine. There is evidence that IL-6 plays a significant role in the acute inflammatory response, escalation of autoimmune reactions, and chronic inflammatory diseases [55–57]. IL-8 is a chemokine and secreted by macrophages. It has been reported that IL-8 is involved in acute inflammation and plays a central role in the initiation and maintenance of the inflammatory response in various inflammatory diseases [58–60]. MCP-1 recruits monocytes and memory T cells to the inflammation area. It has been well documented that MCP-1 mediates both acute and chronic inflammation [61]. MCP-1 has also been shown to stimulate IL-6 secretion and NF-*κ*B activation [62]. One significant finding of this investigation is that IL-6 and -8 and MCP-1 expression was decreased after ANG treatment, indicating that ANG likely participates in attenuation of the inflammatory response through inhibition of TBK1 expression.

IL-4 and -10 are known to suppress the inflammatory response. IL-4 causes vitalization of triggered B-cell and T-cell proliferation, and IL-10 improves B-cell survival and proliferation. It has been suggested that IL-4 and -10 have anti-inflammatory activity and repress the expression of proinflammatory cytokines [63–65]. In this study, we found that ANG treatment increased mRNA expression of both IL-4 and IL-10. It can therefore be presumed that the expression of IL-6 and -8 was downregulated by ANG.

TNF-*α* binds TNFR 1 and 2 and triggers the activation of NF-*κ*B [66]. Phosphorylation of TBK1 and TNFR allows NF-*κ*B nuclear translocation [21], which results in the inflammatory response cascade including amplification of TBK1, IL-6 and -8, and MCP-1 [22, 67, 68]. NF-*κ*B plays an important role in regulating the immune response and inflammation. It has been reported that NF-*κ*B is involved in several inflammatory diseases [69–72]. The activation of NF-*κ*B is inhibited by I*κ*B proteins such as TBK1, which deactivate NF-*κ*B by arresting it in the cytoplasm. Our results suggest that ANG may reduce NF-*κ*B nuclear translocation through inhibition of TBK1 expression and reduction of TNFR1 and 2 mRNA expression.

**Figure 6:**
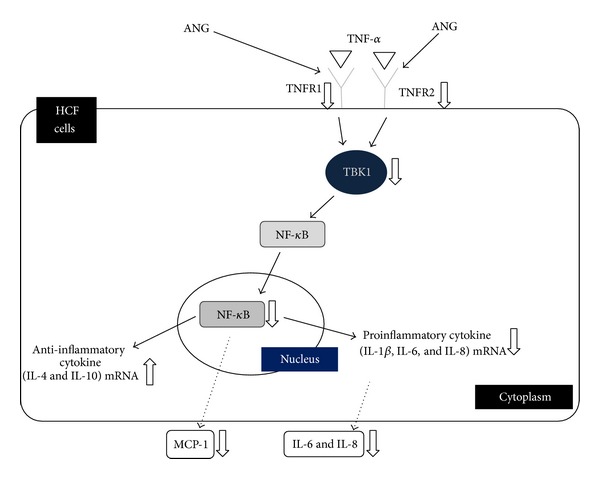
Schematic model illustrating the signaling pathway by which ANG suppresses the inflammatory response involving TBK1-mediated NF-*κ*B nuclear translocation in HCFs inflamed by TNF-*α*. ANG reduces the mRNA expression of proinflammatory cytokines (IL-1*β*, -6, and -8) and enhances the mRNA expression of anti-inflammatory cytokines (IL-4 and -10). ANG also inhibits NF-*κ*B nuclear translocation through a reduction in TNFR1 and 2 mRNA expression and TBK1 production. The cascade underlying the effect of ANG results in a decrease in inflammatory cytokines and chemokines such as MCP-1 and IL-6 and -8.

A schematic illustration of the anti-inflammatory signaling pathway induced by ANG treatment of HCFs inflamed by TNF-*α* is shown in Figure 6. TNF-*α* induces an inflammatory signal by binding TNFR1 and 2. The inflammatory response is mediated by activation of TBK1, which is required for NF-*κ*B nuclear translocation. ANG downregulates mRNA expression of IL-1*β*, -6, and -8. Moreover, ANG upregulates mRNA expression of IL-4 and -10. ANG also inhibits NF-*κ*B nuclear translocation through inhibition of TBK1 production. The anti-inflammatory effect induced by ANG results in a reduction of proinflammatory cytokines and chemokines such as MCP-1 and IL-6 and -8.

Since ANG is an inducer of new blood vessel growth, ANG treatment may possibly generate injection on the ocular surface. Because this study is confined to* in vitro* tests, we cannot exclude the possible adverse effects of ANG* in vivo*. However, this investigation is the first to report the function of ANG in the inflammatory response.* In vivo* animal studies are needed to further validate our hypothesis prior to the clinical application of ANG in ocular inflammation.

There is little information in the literature regarding the anti-inflammatory effects of ANG. The ANG functions newly identified in this study indicate that it likely plays a critical role in attenuating inflammation. The findings pointing to the anti-inflammatory effects of ANG in inflamed HCFs shed new light on the treatment of ocular inflammation. We have demonstrated that ANG suppresses the TNF-*α*-induced inflammatory response and NF-*κ*B nuclear translocation through inhibition of TBK1 expression in HCFs. This preliminary evidence suggests that ANG could be a strong candidate for the treatment of corneal inflammation.

## Supplementary Material

The data about identity of the purified ANG was obtained from the Department of Biochemistry at Chungbuk National University. They had already confirmed the purity and effect of angiogenin by western blotting with ANG specific antibodies in previous article (Srisa-Art M et al. Analysis of protein-protein interactions by using droplet-based microfluidics. Chembiochem : a European journal of chemical biology. 2009). The biological activity of the purified ANG was confirmed by its nuclear translocation in human umbilical vein endothelial (HUVE) cells. The enzymatic activities of the purified ANG toward poly (C) were measured and compared it with RNase A as shown in Supplementary Figure 1. ANG catalyzes the cleavage of poly (C), and the ribonucleolytic activities of ANG were determined by measuring the rate of formation of perchloric acid-soluble products in poly (C) precipitation assay. The endotoxin concentrations in ANG preparation were determined using the Limulus amebocyte lysate (LAL) assay. A standard curve was used to determine the endotoxin quantities presented in the purified angiogenin as shown in Supplementary Figure 2. Endotoxin level was determined to be 0.011 ng per **μ**g of the purified angiogenin (0.11 EU/ **μ**g). The results suggest that the purified ANG has both the biological and enzymatic activities and contains verified low level of endotoxin for its use both in vitro and in vivo experiments.

## Figures and Tables

**Table 1:** PCR primers used in this study.

Gene	Sense primer (5′→3′)	Antisense primer (3′→5′)	PCR product size (bp)
GAPDH	CGAGATCCCTCCAAAATCAA	TGTGGTCATGAGTCCTTCCA	294
IL-1*β*	CCTGTCCTGCGTGTTGAAAGA	GGGAACTGGGCAGACTCAAA	150
IL-4	TGTCTGTTACGGTCAACTCG	ACATTGTCACTGCAAATCGA	195
IL-6	TTCGGTCCAGTTGCCTTCTC	GAGGTGAGTGGCTCTCTGTG	122
IL-8	ACATGACTTCCAAGCTGGCCG	TTTATGAATTCTCAGCCCTC	303
IL-10	GCCTAACATGCTTCGAGATC	TGATGTCTGGGTCTTGGTTC	206
TNFR1	GTGCTGTTGCCCCTGGTCAT	GCTTAGTAGTAGTTCCTTCA	163
TNFR2	AAACTCAAGCCTGCACTC	GGATGAAGTCGTGTTGGAGA	209
TBK1	TTCTGGAAGTCCATACGCAT	ACTGGTGATCTCTATGCTGT	237
