# Supplementary material for: Angiogenin Reduces Immune Inflammation via Inhibition of TANK-Binding Kinase 1 Expression in Human Corneal Fibroblast Cells
Source: Mediators Inflamm. 2014 Apr 17;2014:861435. doi: 10.1155/2014/861435 (PMC4016892; doi:10.1155/2014/861435)
Supplement: Supplementary file 1 — The data about identity of the purified ANG was obtained from the Department of Biochemistry at Chungbuk National University. They had already confirmed the purity and effect of angiogenin by western blotting with ANG specific antibodies in previous article (Srisa-Art M et al. Analysis of protein-protein interactions by using droplet-based microfluidics. Chembiochem : a European journal of chemical biology. 2009). The biological activity of the purified ANG was confirmed by its nuclear translocation in human umbilical vein endothelial (HUVE) cells. The enzymatic activities of the purified ANG toward poly (C) were measured and compared it with RNase A as shown in Supplementary Figure 1. ANG catalyzes the cleavage of poly (C), and the ribonucleolytic activities of ANG were determined by measuring the rate of formation of perchloric acid-soluble products in poly (C) precipitation assay. The endotoxin concentrations in ANG preparation were determined using the Limulus amebocyte lysate (LAL) assay. A standard curve was used to determine the endotoxin quantities presented in the purified angiogenin as shown in Supplementary Figure 2. Endotoxin level was determined to be 0.011 ng per μg of the purified angiogenin (0.11 EU/ μg). The results suggest that the purified ANG has both the biological and enzymatic activities and contains verified low level of endotoxin for its use both in vitro and in vivo experiments. [file 861435.f1.pdf]

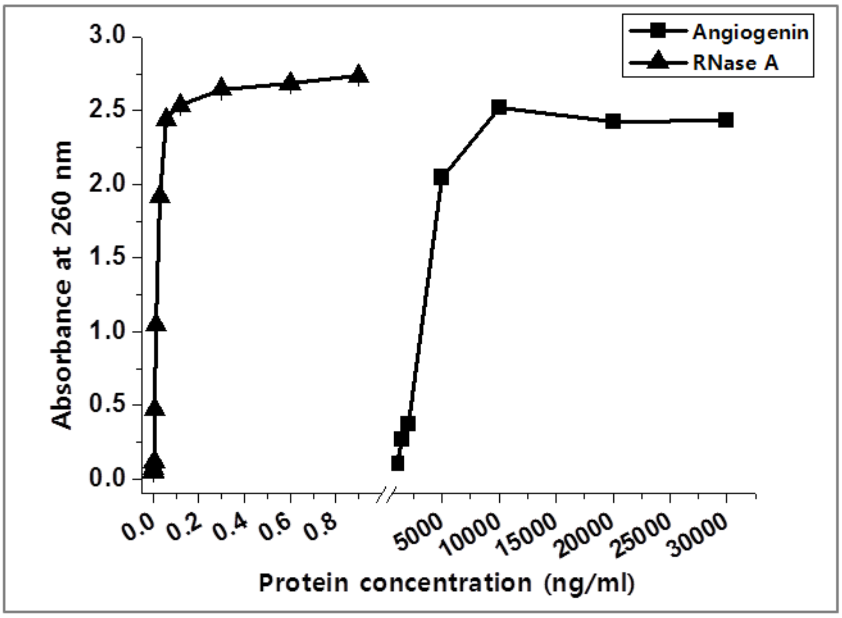

SUPPLEMENTARY FIGURE 1. Poly(C) precipitation assay for the purified ANG. The ribonucleolytic activities of ANG and RNase A were determined by measuring the rate of formation of perchloric acid-soluble products in poly(C) precipitation assay. Briefly, the serial dilutions of ANG or RNase A were incubated with 60 µl of 2 mg/ml poly (C) in 30 mM Hepes pH 7 for 4 hours at 37 ℃. The reaction was terminated by chilling on ice and adding 700 µl of 3.4% cold perchloric acid. After 10 min on ice the samples were centrifuged at 13,000 rpm for 10 min at 4 ℃. The supernatant of each sample was measured at 260 nm. All readings were corrected for the absorbance of blanks that lacked ANG or RNase A. The assays were carried out in duplicate.

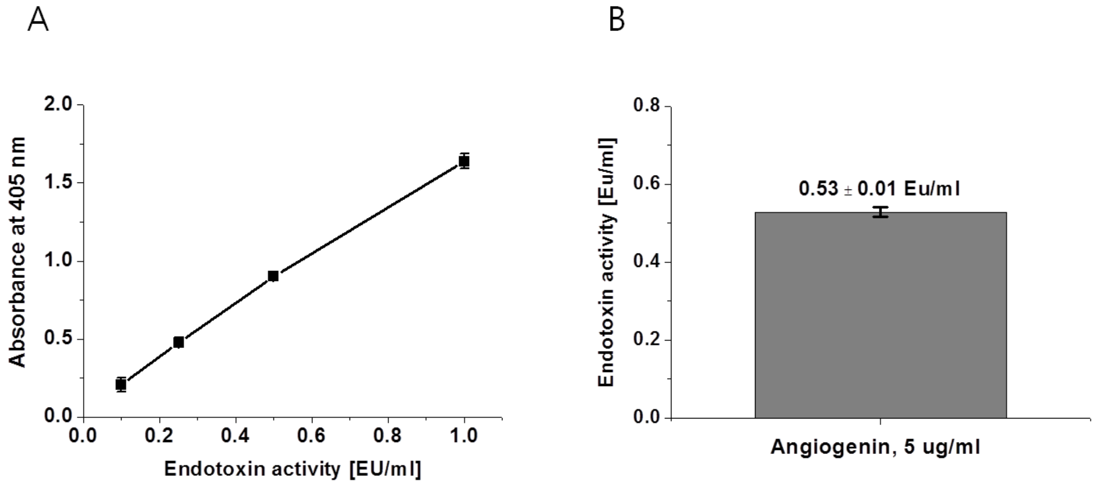

SUPPLEMENTARY FIGURE 2. Endotoxin assay for the purified ANG. (A) A standard curve is created using the E. coli endotoxin standard. One endotoxin unit/mL (EU/mL) equals approximately 0.1 ng endotoxin/mL of solution. Coefficient of determination (R^2) is 0.99. (B) The endotoxin concentrations in the purified angiogenin were determined using a standard curve shown in (A). Endotoxin level of the purified angiogenin is 0.53 EU per mL of solution. Results can be converted to unit of endotoxin per µg of the purified angiogenin (0.11 EU/µg).
